# Editorial: Controlling biofilm-related infections in healthcare settings

**DOI:** 10.3389/fcimb.2025.1679631

**Published:** 2025-08-28

**Authors:** Reham Wasfi, Samira M. Hamed, Mahmoud A. Elfaky

**Affiliations:** ^1^ Department of Microbiology and Immunology, Faculty of Pharmacy, October University for Modern Sciences and Arts (MSA), Giza, Egypt; ^2^ Department of Natural Products, Faculty of Pharmacy, King Abdulaziz University, Jeddah, Saudi Arabia; ^3^ Centre for Artificial Intelligence in Precision Medicines, King Abdulaziz University, Jeddah, Saudi Arabia

**Keywords:** biofilm, antimicrobial resistance, virulence, infections, antibiofilm, treatment

Biofilm formation by clinically significant pathogens presents a substantial global public health challenge, particularly due to its role in enhancing antimicrobial resistance (AMR). The protective matrix of biofilms contributes to bacterial persistence and adaptation, exacerbating multidrug resistance (MDR), particularly in nosocomial infections (Elfaky et al., 2024). These infections result in elevated patient morbidity and mortality and impose considerable economic burdens on healthcare systems due to increased costs and prolonged hospital stays (Assefa and Amare, 2022). Consequently, there has been a marked surge in research aimed at identifying novel strategies to combat biofilm-associated infections (Wasfi et al., 2023). This Research Topic encompasses three critical aspects of biofilm research: the prevalence of biofilm-forming pathogens, the molecular mechanisms underlying biofilm formation, and innovative approaches for combating biofilm-associated infections.

Surveillance studies on the prevalence and resistance patterns of biofilm-forming pathogens are critical for quantifying the magnitude of this threat. The national surveillance study by Thabit et al. aimed to evaluate the antimicrobial susceptibility of *Pseudomonas aeruginosa* isolates collected from diverse clinical sources across seven regions in Saudi Arabia and to identify MDR, difficult-to-treat (DTR), and pandrug-resistant (PDR) strains. All isolates were classified as MDR. Amikacin showed the highest susceptibility (76.8%), while carbapenems showed moderate activity (52%). Alarmingly, colistin showed only 43.8% susceptibility. About 22.2% of isolates were DTR, and 5.4% were PDR. The majority of resistant strains came from respiratory and skin/soft tissue infections. Notably, susceptibility to commonly used antibiotics like piperacillin/tazobactam and ceftazidime was low.

*Staphylococcus aureus* presents another significant threat to vulnerable populations, including hemodialysis patients. In an eight-year study, Lai et al. analyzed 103 *S. aureus* isolates from this patient group. Their work revealed that both methicillin-resistant *S. aureus* (MRSA) and methicillin-sensitive *S. aureus* (MSSA) strains formed strong biofilms, with *agr* type I predominating and *agr* type II strains carrying more virulence genes. The frequent presence of biofilm-associated gene combinations among the isolates highlights the genetic adaptability of *S. aureus*, contributing to its persistence and resistance in device-related infections.

Another notable example of a biofilm-associated pathogen in device-related infections is *Proteus mirabilis*. The review by Yang et al. provides a comprehensive overview of the complex and diverse virulence mechanisms of *P. mirabilis*. Notably, the review is the first to describe the roles of the hydrogenase system, autotransporter proteins, the molybdate-binding protein ModA, and two-component systems as virulence factors in *P. mirabilis*.

In this Research Topic, considerable attention has been given to uncovering the mechanisms driving biofilm formation. Slobodianyk-Kolomoiets et al. explored polymicrobial biofilms in chronic periodontitis. Using confocal laser scanning microscopy, they analyzed subgingival biofilms formed on biologically neutral polyethylene terephthalate films placed in the gingival cavities of patients. This approach allowed for detailed visualization of the biofilm’s extracellular polymeric substances (EPS), including amyloids, proteins, carbohydrates, and extracellular DNA (eDNA). Their findings revealed that eDNA was the predominant matrix component, with the majority derived from the host, likely through neutrophil extracellular traps, rather than from bacterial sources. These results underscore the significant role of host-derived components in biofilm stabilization and highlight the dynamic interactions between immune responses and microbial biofilm development.

The topic also raised an issue where many of the experimental antibiofilm compounds under study lack further tests on their applicability and safety for clinical use, which require further investigations by *in vivo* studies. Among the ESKAPE pathogens of concern, *Enterococcus faecalis* is frequently implicated in persistent root canal infections and is a leading cause of endodontic treatment failure (Siqueira and Rôças, 2022). Despite the promising *in vitro* efficacy of several anti-biofilm interventions, their clinical applicability remains underexplored. Yang et al. highlighted various experimental approaches for targeting *E. faecalis* biofilms, including nanoparticles and bacteriophage therapy. Nanoparticles demonstrate broad-spectrum activity, whereas bacteriophages offer highly specific targeting. The synergistic use of phages and antibiotics may outperform monotherapies. Additionally, phytotherapeutic agents such as trans-cinnamaldehyde, quercetin, and extracts from grape seeds, tea tree oil, *Berberine*, *Aloe vera*, propolis, and *Triphala* have shown anti-biofilm activity. Probiotics also present a promising natural alternative; however, their clinical development necessitates further *in vivo* studies, particularly focusing on innovative delivery systems like biocompatible carriers and sustained-release formulations to enhance efficacy and safety.

Al-Rabia et al. examined the quorum sensing (QS) inhibitory activity of thymoquinone (TQ), a bioactive compound derived from *Nigella sativa*, against *P. aeruginosa*, demonstrating its potential as an effective antivirulence agent. Similarly, Zhang et al. investigated the effects of Tanreqing (TRQ), a traditional Chinese medicinal preparation composed of five herbal constituents, and reported significant inhibition of biofilm formation and QS gene expression in *Klebsiella pneumoniae*. TRQ treatment enhanced bacterial clearance both *in vitro* and *in vivo*, supporting its potential therapeutic utility. Collectively, these studies highlight the promising anti-QS and anti-biofilm activities of phytotherapeutic agents and plant-derived extracts. Moreover, *in vivo* evaluations confirmed the safety and applicability of these natural compounds, indicating their potential for clinical development.

The incorporation of antibiofilm agents into medical devices such as wound dressings and indwelling catheters has emerged as a promising strategy for the localized prevention and disruption of biofilm formation (Amer et al., 2022, 2023). This enables sustained release of active compounds directly at the infection-prone site, thereby enhancing antimicrobial efficacy while minimizing systemic toxicity. *S. aureus*, especially MRSA, is a major causative agent of skin and soft tissue infections (SSTIs), often originating from nasal carriage (Costa et al., 2024). Accordingly, numerous approaches have explored incorporating antibiotics and anti-inflammatory agents into wound dressings and polymeric films to control MRSA infections (Ghataty et al., 2022). Nitric oxide (NO) has emerged as a potent antimicrobial with proven biofilm-disrupting capabilities against both Gram-positive and Gram-negative bacteria (Cui et al., 2024). Due to its short half-life, NO is delivered via hydrogels, ointments, and nanoparticles for controlled release (Choi et al., 2020). The study by Davis et al. aimed to evaluate the antimicrobial effectiveness of nitric oxide topical formulations against MRSA strains isolated from nasal colonization using a porcine wound infection model. The primary objective was to assess the efficacy of different concentrations of a NO-releasing ointment in reducing MRSA biofilm burden in deep partial-thickness wounds infected with a clinical nasal MRSA isolate. The 1.8% NO formulation significantly reduced MRSA burden, achieving over 99% bacterial reduction, closely matching the efficacy of Mupirocin 2%. Lower concentrations (0.3% and 0.9%) also demonstrated statistically significant bacterial reductions, though less effective than the highest concentration. All NO treatments showed a downward trend in bacterial load from day 4 to 7.
